# Clinician and simulated patient perspectives on ambient AI scribes in psychiatric consultations: a qualitative study

**DOI:** 10.3389/fpsyt.2026.1821065

**Published:** 2026-05-28

**Authors:** Syed Ali Bokhari, Faisal A. Nawaz, Firdous M. Usman, Zara Arshad, Meghana Sudhir, Ralf Krage, Syed Fahad Javaid, Rahul Kashyap

**Affiliations:** 1 Al Amal Psychiatric Hospital, Emirates Health Services, Dubai, United Arab Emirates; 2 Global Remote Research Scholars’ Program, Princeton Junction, NJ, United States; 3 Department of Oncology, American Hospital Dubai, Dubai, United Arab Emirates; 4 Institute of Learning, Mohammed Bin Rashid University of Medicine and Health Sciences, Dubai, United Arab Emirates; 5 Department of Psychiatry, College of Medicine and Health Sciences, United Arab Emirates University, Al Ain, United Arab Emirates; 6 Department of Anesthesiology and Critical Care Medicine, Mayo Clinic, Rochester, MN, United States; 7 Department of Research, WellSpan Health, York, PA, United States

**Keywords:** AI in psychiatry, ambient AI, artificial intelligence, clinician burnout, digital mental health, patient experience, psychiatric documentation, qualitative research

## Abstract

**Background:**

Documentation burden contributes significantly to psychiatric clinician burnout, with psychiatrists spending an average of three hours per workday on administrative tasks rather than direct patient care. While quantitative studies demonstrate that ambient artificial intelligence (AI) scribes reduce workload and improve documentation quality, the experiential perspectives of clinicians and standardized patients (SPs) remain unexplored, particularly in psychiatry where the therapeutic relationship is central to practice. Our aims were to explore clinician and standardized simulated patient perspectives on ambient AI scribes in psychiatric consultations, examining experiences, concerns, and conditions for successful implementation.

**Methods:**

This study was part of a cross-sectional quantitative simulation study, where qualitative part was planned a-priori. We conducted focus groups with psychiatrists and SPs following a simulation-based crossover study comparing traditional versus AI-assisted documentation. Data were analyzed using Braun and Clarke’s reflexive thematic analysis framework.

**Results:**

In this focus group session with eight psychiatrists and four SPs, total six themes were identified: (1) Clinical Presence, whereby reduced documentation barriers were perceived to allow more authentic human connection; (2) Liberation from documentation burden, with clinicians describing cognitive and emotional relief beneficial for their mental health; (3) Clinical Competence Amplified, in which clinicians perceived the AI scribe as enhancing practice through intelligent translation to psychiatric terminology and prompting for missed assessments; (4) Calibrating Trust, where participants navigated initial uncertainty while recognizing the importance of human oversight; (5) Privacy, stigma, and psychiatric exceptionalism, highlighting unique consent and confidentiality considerations for psychiatric populations; and (6) Perceptions of an enhanced standard of care, with both groups expressing strong preference for AI-assisted consultations with conditions for adoption.

**Conclusion:**

In this exploratory simulation-based qualitative study, AI scribes were perceived as having potential to support more present, patient-centered psychiatric consultations by reducing documentation burden. Implementation should be cautious and requires transparent consent processes, clinician training in behavioral adaptations, robust human oversight, specialty-specific templates, and real-world evaluation with patients receiving psychiatric care.

## 1 Introduction

Documentation demands represent a significant contributor to clinician burnout in psychiatry. Studies report that psychiatrists spend an average of three hours per workday on documentation rather than direct patient care (1, 2), with evidence suggesting that physicians spend approximately two hours documenting for every hour of patient care (3). This administrative burden not only affects clinician well-being but may compromise the therapeutic relationship central to psychiatric practice, where patients require clinicians who are fully present rather than divided between the patient and the screen (4). The quality of the therapeutic alliance, which encompasses the collaborative relationship between clinician and patient including agreement on treatment goals, agreement on therapeutic tasks, and the development of a bond, is a robust predictor of outcomes across a range of mental health treatments (5, 6).

Ambient artificial intelligence (AI) scribes have emerged as a promising solution, using natural language processing and large language models to automatically generate clinical documentation from consultation dialogue (7). Quantitative studies across medical specialties have demonstrated reductions in documentation time, cognitive workload, and after-hours charting, alongside improvements in clinician satisfaction (8–10). A Stanford pilot implementation found significant reductions in physician task load and burnout, with improvements in perceived usability (11). A recent simulation-based crossover study among psychiatrists found that AI scribe use was associated with substantially lower workload, improved documentation quality and enhanced clinician satisfaction (12).

However, quantitative metrics alone cannot capture the nuanced experiential dimensions of AI-assisted documentation in psychiatric practice. Psychiatry differs from other medical specialties in its reliance on verbal and non-verbal communication, the sensitive and stigmatized nature of disclosures, and the variable capacity of patients to provide informed consent (13). The decision-making ability of psychiatric patients can be affected as a result of deficits in mental abilities due to impairments in attention, mood, understanding, and reasoning (14). Understanding how clinicians and patients, including simulated patient participants in early-stage evaluation studies, experience AI scribes, their concerns, adaptations, and preferences, is essential for thoughtful implementation.

While qualitative studies have explored physician perspectives on AI scribes in primary care and other specialties (15, 16), Shah and colleagues’ qualitative study physician interviews found that ambient AI scribes positively impacted physician workload, work-life integration, and patient engagement, with physicians reporting that AI scribes enabled ‘face-to-face’ connection with patients (16). Yet the unique relational and ethical considerations of mental health practice remain unexplored.

This study aimed to explore clinician and standardized simulated patient (SP) perspectives on ambient AI scribes in psychiatric consultations, using focus group discussions following a simulation-based crossover study. Given the novelty of the technology in psychiatric practice and the controlled, simulation-based nature of the design, the study is positioned as exploratory and is intended to inform the questions, hypotheses, and safeguards that should shape subsequent evaluation in real-world clinical settings.

## 2 Methods

### 2.1 Study design

We conducted a qualitative descriptive study using focus group methodology to explore clinician and standardized simulated patient perspectives on AI-assisted documentation in psychiatric consultations. This study was nested within a cross-sectional, crossover, quantitative simulation study comparing traditional keyboard-based documentation with ambient AI scribe assisted documentation (12). Ethical approval was obtained from the Dubai Scientific Research Ethics Committee (DSREC; MBRU IRB-2025-203).

### 2.2 Setting and context

The study was conducted at an academic simulation center at Mohammed Bin Rashid University of Medicine and Health Sciences (MBRU), Dubai, United Arab Emirates, in March 2025. Participants had completed a simulation circuit in which each clinician conducted psychiatric consultations using both traditional documentation (typing during or after consultation) and AI scribe assisted documentation (ambient recording with automated note generation).

### 2.3 Ambient AI scribe system

During AI-assisted consultations, the system captured clinician–patient dialogue through an omni-directional microphone, performed automatic speech recognition, and generated an editable draft consultation note through natural language processing and large language model algorithms. Clinicians selected a standardized psychiatric documentation template from within the platform prior to each consultation; this template was aligned with the bio-psycho-social model of psychiatric formulation and was modified for the study to ensure consistency in documentation structure and clinical comprehensiveness across consultations. Clinicians were permitted to edit the generated draft but were instructed not to rewrite the entire note. Audio and transcribed content were processed in accordance with best practice guidelines.

### 2.4 Participants

Two focus groups were conducted. The clinician focus group comprised eight psychiatrists (four female, four male) from a tertiary psychiatric hospital in Dubai. The cohort included three consultants, two specialists, and three residents, all with a minimum of two years of clinical experience. This was their first experience using AI scribe technology. The standardized patient (SP) focus group included four trained standardized simulated patients who had portrayed psychiatric scenarios including obsessive-compulsive disorder, schizophrenia, bipolar disorder (manic episode) and major depressive disorder, completing multiple consultations with different psychiatrists in both documentation conditions. Informed written consent was obtained from participants in both groups prior to the focus group discussions.

### 2.5 Data collection

The clinician focus group was conducted in-person immediately following the simulation circuit (duration: 61 minutes). The SP focus group was conducted virtually via Zoom the same week (duration: 72 minutes). Both discussions were audio-recorded and transcribed verbatim.

A semi-structured interview guide was developed to explore participants’ experiences with AI-assisted documentation compared to traditional methods. The guide addressed five primary domains: (1) overall experience with AI-assisted versus traditional documentation practices; (2) perceived differences in consultation quality and therapeutic interaction; (3) concerns regarding documentation accuracy, privacy, and trust; (4) behavioral or communicative adaptations made during AI-assisted consultations; and (5) preferences and recommendations for future clinical practice. Open-ended probing questions were used throughout to elicit specific examples and encourage elaboration on initial responses.

### 2.6 Data analysis

Reflexive thematic analysis was conducted following Braun and Clarke’s six-phase framework (17, 18): (1) familiarization through repeated reading of transcripts; (2) systematic generation of initial codes across both datasets; (3) searching for themes by collating codes into candidate themes; (4) reviewing themes against coded extracts and full dataset; (5) defining and naming themes; and (6) producing the report.

Transcripts were coded independently by two researchers (SAB and FAN); coding frames were then compared in consensus meetings and any disagreements reconciled through discussion, with unresolved differences adjudicated by a third senior researcher (RK). Themes were constructed collaboratively through iterative review of coded extracts against the full dataset, with data sufficiency judged to be reached when further review yielded no additional codes relevant to the research questions. Reflexivity was maintained throughout, with explicit team discussion of prior involvement in, and awareness of, the positive findings of the parent quantitative simulation study.

Codes and themes were developed iteratively, with attention to both convergence and divergence between stakeholder groups. Quality was assessed using a 16-item checklist for thematic analysis (19).

## 3 Results

**Figure 1:**
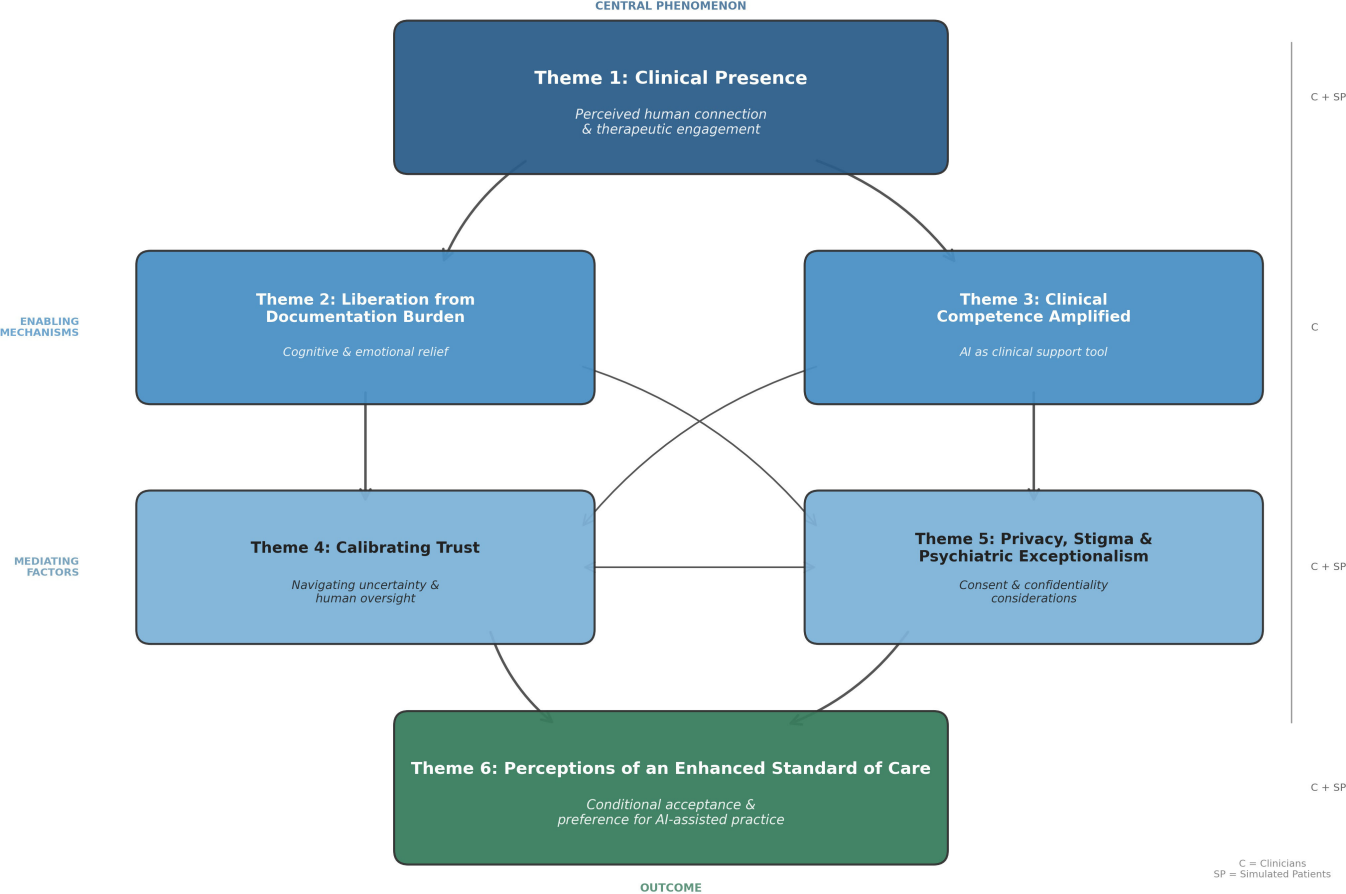
Thematic map of clinician and standardized simulated patient perspectives on ambient AI scribes in psychiatric consultations. The map presents the six themes and their inferred relational structure. Theme 1 (Clinical Presence) is positioned as the central phenomenon, Themes 2 and 3 as enabling mechanisms, Themes 4 and 5 as mediating factors, and Theme 6 as the outcome. C, Clinicians; SP, Standardized Simulated Patients.

Analysis of the focus group transcripts revealed six interconnected themes capturing how clinicians and SPs described the experience of AI-assisted psychiatric consultations (**Table 1**; **Figure 1**). The themes are presented below, beginning with the central phenomenon of clinical presence, followed by the mechanisms that enable it, and concluding with considerations for implementation.

**Table 1:** Summary of themes identified from clinician and standardized simulated patient focus groups.

Theme	Description	Primary source
1. Clinical presence	Transformation of consultation quality through removal of documentation barrier	Clinicians and SPs
2. Liberation from documentation burden	Cognitive and emotional relief enabling clinical focus	Clinicians
3. Clinical competence	AI as clinical support tool enhancing practice quality	Clinicians
4. Calibrating trust	Navigating initial uncertainty while maintaining human oversight	Clinicians and SPs
5. Privacy, stigma, and psychiatric exceptionalism	Unique consent and confidentiality considerations for psychiatry	Clinicians and SPs
6. Perceptions of an enhanced standard of care	Conditional acceptance and enthusiasm for AI-assisted practice	Clinicians and SPs

Themes were generated inductively from codes developed independently across the clinician and SP datasets by two researchers (SAB and FAN). Coding frames were compared in consensus meetings and any disagreements reconciled through discussion, with unresolved differences adjudicated by a third senior researcher (RK). Codes were then clustered into sub-themes and into the six overarching themes shown.

### 3.1 Theme 1: clinical presence

The most prominent theme across both focus groups was a marked shift in the quality of the doctor-patient encounter when the AI scribe was used. Participants described moving from fragmented, divided-attention consultations toward more attentive human engagement. This theme emerged as central to the analysis, with other themes representing either the mechanisms participants felt enabled this shift or the conditions required for its realization.

For clinicians, the experience of undivided attention was revelatory. One psychiatrist articulated the core issue facing the specialty: *‘It is really about the doctor-patient relationship. Patients come to us, especially in psychiatry. They don’t want to see a doctor who’s just busy on the note or looking at the screen.’* This sentiment was echoed by colleagues who described the AI-assisted consultation as liberating them from the perpetual tension between attending to the patient and capturing information. As another clinician expressed, the technology allowed them to *‘forget paper, forget computer, forget everything’* and simply be present with the patient. Another described being able to engage in the kind of spontaneous, humanizing interaction that documentation demands typically preclude: *“I was able to joke around with my patient during this time. She said that she would have wanted to be a doctor or engineer, but she ended up being a graphic designer. I was like, graphic designer is very cool.”*

The same clinician noted that emotionally significant moments, such as when *‘a patient starts crying and I’m consoling the patient’*, could now occur naturally without the intrusion of documentation, and importantly, such therapeutic exchanges were appropriately filtered from the clinical note.

The accounts of the SPs corroborated the clinicians’ experiences. As trained standardized patients rather than individuals with psychiatric illness, their reports are best read as informed observations of the encounter rather than lived patient experience. Without knowing what clinicians had reported, they independently identified the same shift. Participant SP1, who portrayed a patient with obsessive-compulsive disorder, described the difference in eye contact: *“When it was the AI, there was more eye-to-eye contact, and they could pick up on the same actions that I was doing.”* Participant SP2, who portrayed a patient with schizophrenia, similarly observed feeling *“more attentive when doctors just casually talking face to face and rather not making notes at the same time.”* The metaphor of friendship emerged spontaneously in the patient focus group, with SP1 noting that “*10 minutes into the consultation it was like talking to maybe like a friend … there was not this thing where they had to type on computer.*”

Perhaps most clinically significant was the observation by SPs that non-verbal cues, which are critical in psychiatric assessment, were detected more rapidly during AI-assisted consultations. SP1, whose OCD scenario included characteristic picking behaviors, noticed a striking difference: *“When the psychiatrist was actually typing … it took them longer to notice those things. But when it was the AI, it was picked up on the first time the first moment I started picking something on my dress it was noticed. In the traditional I had to pick a lot more for it to be noticed.”* The closing of consultations also differed, with SPs describing AI-assisted endings as “more relaxed” rather than rushed, allowing space for questions and a proper therapeutic conclusion.

### 3.2 Theme 2: liberation from documentation burden

While Theme 1 describes the transformation experienced in the consultation room, Theme 2 illuminates the mechanism from the clinician’s perspective: the removal of a cognitive and emotional burden that had been constraining practice. Clinicians spoke not merely of time savings but of a fundamental shift in their experience of clinical work.

The language clinicians used was striking in its reference to wellbeing and mental health, their own. One psychiatrist stated directly: *“This is very helpful for the mental health of the psychiatrist.”* This framing of documentation burden as a threat to physician mental health reframes the issue from one of efficiency to one of occupational wellbeing. Colleagues elaborated on the specific burdens lifted: *“I tend to forget to put in notes or delay notes because that’s a pressure on my mind.”* Another clinician described the relief in terms of both physical and cognitive load: *“The physical load of doing anything was not there. And the fact that I have to remember all the conversation that I was asking, a whole lot of questions was also not there.”*

This cognitive offloading had immediate clinical benefits. One participant described finally being able to apply training that documentation demands had previously precluded: *“When I was doing the AI interview, I had more time to go with more thorough history. I was able to ask about trauma history, past trauma, I was able to ask about pre-morbid personality.”* Another made a poignant observation about examinations versus clinical practice: *“I really felt that I’m doing my exam thing (knowledge from examinations) because now I was able to put whatever I’ve learned for examination, into practice for the first time.”* The implication that routine clinical practice fails to match the standard expected in examinations, specifically because of documentation demands, points to a perceived compromise of clinical quality because of time constraints that AI scribes may help address.

### 3.3 Theme 3: clinical competence

Beyond removing barriers, clinicians described the AI scribe as potentially enhancing their quality of care.

A particularly valued capability was the AI’s translation of colloquial patient language into appropriate psychiatric terminology. One clinician illustrated this with an example from their consultation: *“I’m not going to ask the patient ‘are you having auditory hallucinations’, but it still translated it as ‘denied auditory hallucinations’, even though my question was ‘could you hear any voices’. So that was a very good interpretation of the psychiatric terms.”* This interpretation was perceived as serving multiple functions: it allows clinicians to maintain natural, patient-centered language during consultations while still generating documentation that meets professional standards and facilitates communication with colleagues.

However, this translation also raises a concern: by documenting that the patient “denied auditory hallucinations”, the AI may imply that the clinician explicitly assessed for this symptom, when in fact the question may not have been asked in that specific form, or at all. This distinction between translating what was discussed and inferring what was not is an important accuracy consideration, as AI systems can generate entirely fictitious content, such as documenting examinations that never occurred or creating non-existent diagnoses (7, 20).

Clinicians also discovered an unexpected safety-net function. The AI scribe’s documentation template highlighted areas of the assessment that were not discussed. One participant noted: *“It did mention that forensic history was not assessed. So that kind of is something that might be useful in my real-life practice.”* Another described their reaction to seeing ‘not done’ annotations: *“The points that I did not do were mentioned, ‘not done.’”* Risk assessment was specifically mentioned as an area where such a feature could be valuable. The structured output, described as *“starting from history, mental state examination, diagnosis, everything, my plan”*, was also noted to capture elements that clinicians acknowledge often omitting from traditional documentation, particularly detailed management plans and formulation. One clinician estimated that *“90% of workload is being done by AI, and then 10% requiring editing”*, representing a substantial redistribution of documentation effort.

Importantly, clinicians observed that the AI appropriately filtered non-clinical content. Therapeutic exchanges, humor, and emotionally supportive statements were not incorporated (appropriately so) into the clinical record, preserving both the professionalism of documentation and the authenticity of the therapeutic encounter.

### 3.4 Theme 4: calibrating trust

The benefits described in the preceding themes were not experienced without complexity. Both clinicians and SPs described a process of calibrating trust in the AI system, navigating initial uncertainty while developing an understanding of appropriate human oversight.

Clinicians were candid about initial anxiety. Accustomed to the tangible security of contemporaneous notes, trusting an invisible system required a leap of faith: *“I was kind of anxious and I’m used to making notes. I’m like, okay, is it actually doing its job? So there was a little bit of anxiety there.”* Concerns about accuracy and capture were common: *“Is it catching everything? How clear is my microphone that it would actually get things?”* One clinician noted observing instances of AI ‘hallucination’, with documentation of clinical findings that had not occurred, such as recording affect as ‘euphoric’ when it was not.

Rather than undermining confidence entirely, however, some clinicians reframed these limitations constructively: *“It’s a good thing that it does not work 100% accurately because I think if it does after ten, twenty sessions, people would be confident in it, people would over rely on it.”* This sophisticated perspective, recognizing that imperfection serves as a safeguard against complacency, suggests clinicians are developing nuanced frameworks for human-AI collaboration. One participant articulated a vision for this partnership: *“AI will not replace humans or physicians. AI plus physicians will replace physicians.”*

Clinicians also described spontaneously adapting their behavior to optimize AI capture. One participant realized they had begun verbalizing observations of non-verbal behavior: *“I was like, ‘I can see that you’re fidgeting while you’re sitting right now.’ So I was trying to describe a thing in that way so that the AI would [capture it] … You have to kind of realign how you’re verbalizing your content.”* Practical concerns about technical reliability and real-world conditions also emerged. One clinician voiced a ‘scary thought’ about system glitches erasing recordings when no backup notes existed. This raised a practical implementation dilemma: whether recordings should be retained as a backup to protect against data loss, or minimized to reduce privacy and confidentiality risks. Others noted that the simulation’s quiet environment differed from typical clinical settings, raising questions about performance amid background noise and family members present for the collateral history. Further testing AI scribe in real clinical settings is warranted.

### 3.5 Theme 5: privacy, stigma, and psychiatric exceptionalism

Both focus groups identified considerations that may be unique to or amplified in psychiatric practice. The intersection of mental health stigma, variable patient capacity, and the sensitive nature of psychiatric disclosure creates a constellation of challenges requiring specialty-specific attention.

SPs emphasized the need for detailed, transparent information before AI documentation is implemented. SP1 reflected on what a real patient might require: *“If it was a real doctor, and if my doctor actually told me that I’ll be recording this session … I would have asked more questions like, what is this? Where is this information going? what is the purpose of this?”* The suggestion of pre-consultation educational materials, such as pamphlets emerged as a practical recommendation from this group. Notably, clinicians acknowledged that consent practices during the simulation had been variable: when asked how many had sought permission to record, only two of eight indicated they had done so, highlighting an implementation gap.

The stigma associated with psychiatric consultation added a layer of complexity not typically present in other medical specialties. SP4, drawing on her experience portraying a patient with depression, observed: *“One of my characters, she was worried about how her in-laws are going to say if they know that she went to see a psychiatrist … Sometimes patients worried about other people knowing they went to see a psychiatrist.”*

Perhaps most significantly for psychiatric practice, questions emerged about capacity to consent among SPs with impaired mental states. SP2, who portrayed a patient with schizophrenia, offered a perspective from within that role: *“As a schizophrenia patient, if I have been asked to give consent for AI, maybe I will not.”* SP4 elaborated on the capacity issue: “I don’t know how much they can understand that if they are not in a clear mind state.” These observations suggest that standard consent processes may be insufficient for psychiatric populations and that modified approaches, perhaps involving capacity assessment or surrogate consent for AI use may be necessary.

SPs drew a clear and reassuring boundary regarding the scope of AI involvement. SP2 articulated this distinction: *“For documentation, I’m okay, 10 on 10, I have no problems. But then when it comes to diagnosis, medication, I would love to know how much the doctor is relying on that.”* This delineation, showing comfort with AI for transcription while requiring assurance about the primacy of human clinical judgment, provides guidance for how AI scribes should be positioned in patient communications. As ambient AI documentation tools move toward clinical implementation, appropriate disclosure during clinician-patient encounters will require explicit procedural guidance.

### 3.6 Theme 6: perceptions of an enhanced standard of care

Despite the concerns and conditions articulated in the preceding themes, both clinicians and SPs expressed clear preference for AI-assisted consultations. This final theme captures the sense that participants had glimpsed a potentially preferable model of documentation-supported psychiatric care.

Clinicians’ endorsement was notable for acknowledging limitations while still affirming overall preference: *“Despite all the negatives we mentioned, I would still want to use it. I would definitely still want to use it. It does help. It makes your work easier.”* SPs articulated their preference through the lens of what mattered most to them: time and attention. SP4 stated simply: *“If the doctor can spend more time with me since the AI is documenting things, then I would prefer the doctor with an AI-assisted things.”* SP3 was more emphatic: *“Being a patient, for me, the AI assistant was way better than the traditional one.”* When asked to summarize the benefit of AI scribes in psychiatric consultations in a single word, SP1 responded: *“Patient-centeredness.”* This encapsulation, from the patient perspective, suggests that the perceived value of the technology lay in its potential to return attention to the patient within the consultation.

Clinicians’ enthusiasm was tempered by specific conditions for adoption. Customizability emerged as important, with one clinician expressing concern about loss of individual clinical voice: *“If the system is not customizable, if it doesn’t learn my style, I won’t feel very comfortable about it … The individuality or the humanness or the uniqueness of each psychiatrist gets out the window. It makes everything very systematic and technically like robots, every physician becomes the same.”* This tension between standardization and individualization represents an important consideration for implementation. Clinicians also expressed aspirations for integration with electronic medical records and longitudinal capture of patient history, suggesting they envision AI scribes as part of an evolving documentation ecosystem rather than a standalone tool.

### 3.7 Divergent perspectives and negative cases

Not all participants experienced AI scribes as transformative, and attention to divergent cases provides important nuance to the findings. One clinician reported no change in empathic engagement between conditions: *“For me, I didn’t feel, from my perception of the patient, a difference in either situation … the empathetic output didn’t change for me.”* This suggests that the benefits of AI scribes may be more pronounced for clinicians who experience greater divided-attention burden under traditional documentation, while those who already maintain consistent engagement may see less dramatic improvement.

Another clinician described AI scribe as situationally rather than universally beneficial: *“When I’m more rested, I might actually go for the traditional one. When I’m piled up on work, probably the AI might be useful.”* This perspective positions AI scribes as a tool for high-demand periods rather than a replacement for traditional practice, which may inform flexible implementation models.

Within-participant ambivalence was also notable. Some clinicians who expressed strong overall preference for AI-assisted consultations simultaneously voiced specific reservations, including the concern that non-customizable outputs could erode individual clinical voice and the reflection that AI imperfection was ‘a good thing’ because complete reliability would invite overreliance. SPs similarly moved between enthusiasm for the documentation benefits and clear limits on where AI involvement should stop, as illustrated by SP2’s sharp distinction between comfort with AI transcription and unease about AI contributing to diagnosis or medication decisions. These divergent and ambivalent voices temper a reading of the data as uniformly positive and indicate that any implementation will need to accommodate a spectrum of clinician and patient responses rather than a single dominant narrative.

## 4 Discussion

This exploratory qualitative study offers the first in-depth account of clinician and standardized patient perspectives on ambient AI scribes in simulated psychiatric consultations.

### 4.1 Presence and the therapeutic relationship

Participants’ accounts of restored clinical presence and a stronger sense of therapeutic relationship are particularly significant for psychiatry. Unlike other medical specialties where physical examination and investigations contribute substantially to diagnosis, psychiatric assessment relies heavily on verbal exchange, observation of non-verbal cues, and the quality of rapport (13). Evidence consistently demonstrates that the therapeutic alliance is a robust predictor of outcomes across a broad spectrum of psychotherapy types, treatment modalities, presenting problems, and contexts (5, 6).

This finding extends previous qualitative work. Shah et al. (2025) reported that primary care physicians valued AI scribes for enabling ‘face-to-face’ connection with patients, identifying positive impacts on physician workload, work-life integration, and patient engagement (16). Our findings suggest that this perceived benefit may be amplified in psychiatry, where SPs described faster recognition of non-verbal cues when clinicians were not distracted by typing. The patient observation that the consultation felt like talking to ‘a friend’ after 10 minutes suggests that the technology may support the development of therapeutic alliance, which has been shown to predict treatment outcomes even in psychopharmacological treatment (21). Because the patient voice in this study is that of trained SPs, these relational observations should be interpreted with caution; they indicate what a professionally informed observer noticed about the encounter rather than how patients with active psychiatric illness would experience an AI-assisted consultation.

### 4.2 Clinician well-being

The theme of ‘liberation from documentation burden’ resonates with growing recognition of documentation as a key driver of clinician burnout (3, 22). Research indicates that electronic health record (EHR) use is perceived as a significant contributor to physician burnout, with substantial clerical and data-entry burdens creating new stressors for healthcare providers (23, 24). Notably, our participants framed this not merely as time savings but as beneficial for their ‘mental health as psychiatrists’, a striking acknowledgment of the emotional toll of competing demands. This language suggests that AI scribes may address not just efficiency but clinician wellbeing. This is supported by emerging evidence: a multicenter quality improvement study across six US health systems found that after 30 days of ambient AI scribe use, the proportion of clinicians experiencing burnout decreased significantly from 51.9% to 38.8%, with significant improvements in cognitive task load and after-hours documentation time (25). Such findings underscore that the benefits observed in our study, where clinicians framed documentation burden as a threat to their own mental health, may translate into measurable reductions in professional burnout, with implications for workforce retention in psychiatry.

The finding that clinicians felt able to apply their training ‘for the first time’ when freed from documentation is both encouraging and concerning: encouraging that the technology enables fuller expression of clinical competence but concerning that current documentation practices may significantly constrain clinical practice. This aligns with research suggesting that EHR burden has created or exacerbated traditional stressors, with portions of documentation requirements involving issues such as billing, quality metrics, and compliance that may appear beyond fundamental patient care (24). Compounding this, a scoping review on clinician competencies for AI-based tools found that despite a rapidly expanding landscape of AI applications in healthcare, very few studies have addressed the specific competencies clinicians need to use such tools effectively, highlighting a critical preparedness gap that may hinder adoption (26).

### 4.3 Clinical quality and safety

Theme 3 highlighted a perceived function of AI scribes as clinical support tools. The prompting for missed assessments (forensic history, risk assessment) represents a potential safety net that could reduce errors of omission. However, participants also raised concerns about anchoring bias, that AI summaries might unduly influence clinical reasoning. The clinician observation that ‘10 people with depression are 10 different people’ highlights the importance of maintaining individualized formulation despite standardized documentation. These findings align with broader discussions about AI transparency and the need for clinicians to understand both the capabilities and limitations of AI tools (27).

The finding that AI scribe accurately translated colloquial language (‘could you hear any voices?’) into psychiatric terminology (‘denied auditory hallucinations’) without losing meaning suggests sophisticated natural language processing. However, the reported instances of ‘hallucination’ (e.g., documenting affect as ‘euphoric’ when it was not) underscore the essential role of clinician review. Research evaluating large language models for clinical note generation has reported hallucination rates of approximately 1.5% and omission rates of 3.5% across nearly 13,000 clinician-annotated sentences (28), whilst a recent commentary noted that modern ambient AI scribes report overall error rates of approximately 1–3%, introducing distinct failure modes including fabrications, omissions, and contextual misinterpretations that create new safety challenges (20). These findings align with concerns raised in the literature about AI accuracy and the need for human oversight (7).

A further concern with prolonged use is the potential for overreliance or automation complacency, in which clinicians progressively accept AI-generated summaries with reduced critical scrutiny; early evidence from adjacent specialties suggests a measurable deskilling effect following sustained AI exposure, including reductions in unassisted diagnostic performance after withdrawal of the tool (29). This risk may be greater among trainees, whose formulation skills are still developing and who may have limited exposure to traditional note-writing before AI-assisted practice becomes routine. Related to this, AI-generated narrative summaries may subtly shape clinical reasoning through anchoring on the first documented impression, narrowing the differential, or importing standardized phrasing that does not fully represent the clinician’s own formulation; recent comparative evidence indicates that large language models can match or exceed physician performance on clinical reasoning tasks, which raises questions about how such outputs should be positioned within rather than substituted for clinician judgement (30, 31). Over longer timeframes, the cumulative effect of AI-generated entries across a patient’s record raises unresolved questions about the longitudinal coherence of documentation, the reproduction of AI phrasing across multiple encounters, and the implications for medico-legal defensibility, clinical audit, and downstream research using these records.

### 4.4 Trust and adaptation

The spectrum of trust-related experiences described by participants, from initial anxiety through healthy skepticism to adaptive behavior, offers considerations for implementation. The reframing of AI imperfection as beneficial (because it prevents over-reliance) represents a sophisticated understanding of human-AI collaboration. Research on informed consent for ambient AI documentation has similarly emphasized that patient comfort varies based on trust in their clinician, understanding of the tool, and perceived benefits or risks (32, 33). The behavioral adaptations described, such as verbalizing observations of non-verbal cues, suggest that clinicians naturally develop strategies to optimize AI performance, which could inform training recommendations.

### 4.5 Psychiatric exceptionalism

Theme 5 identifies considerations that may be unique to or amplified in psychiatric practice. The intersection of stigma, capacity concerns, and sensitive disclosure creates a constellation of challenges for AI documentation in mental health. The patient observation that a schizophrenia patient might decline AI consent, and the concern about capacity to consent when ‘not in a clear mind state’, highlight the need for modified consent processes for psychiatric populations. Research has demonstrated that decision-making capacity varies considerably among psychiatric inpatients, with roughly 50% of patients hospitalized with acute schizophrenia showing impairment in at least one element of competence (14, 34).

The clear boundary drawn between AI for documentation versus diagnosis is reassuring but requires explicit communication. SPs were comfortable with AI transcription but wanted assurance that clinical judgment remained with the physician. This finding aligns with broader research on patient perspectives regarding AI in healthcare, which has noted desires for transparency in algorithm credibility and insights into algorithm reliability and accuracy, with particular concerns when AI extends beyond documentation to clinical decision-making (32, 35).

Three further considerations warrant emphasis. First, capacity to consent in psychiatric populations is not a one-off determination but may fluctuate across and even within consultations, particularly in acute psychosis, mania, or severe depression (38); consent obtained at the start of a contact may no longer reflect the patient’s position later in the same encounter, raising practical questions about whether ambient recording should be paused or re-consented at phase transitions, and whether capacity for consent to AI documentation should be assessed separately from capacity for consent to treatment. Second, ambient systems will inevitably capture disclosures of a particularly sensitive nature, including suicidal ideation, trauma histories, abuse, and forensic material; the tension between clinically necessary capture, medico-legal record-keeping, and the patient’s reasonable expectation of discretion in mental health settings is unresolved (33, 36), and was reflected in SPs’ distinction between comfort with AI for documentation and unease about what would be stored and where. Third, generic ethical frameworks for clinical AI offer limited psychiatry-specific guidance on these points (39); the broader debate on physician autonomy under AI augmentation has only recently begun to address mental-health-specific dimensions (37), and local policy development should involve patients with lived experience alongside clinicians and regulators.

### 4.6 Strengths and limitations

Strengths of this study include the dual-stakeholder design capturing both clinician and standardized simulated patient perspectives, the use of established thematic analysis methodology, and the integration with quantitative findings from the parent study. The focus group format enabled rich discussion and elaboration of perspectives. Attention to divergent perspectives and within-participant ambivalence strengthened the analytic rigor, while reflexive acknowledgment of the team’s prior knowledge of positive quantitative findings enhanced transparency.

Limitations of this study are substantial and position the work as exploratory rather than confirmatory. The sample was modest and drawn from a single center in the UAE, limiting transferability. The simulated environment cannot fully replicate the emotional intensity, ambient noise, competing demands, family involvement, or sustained workload of real psychiatric consultations, and benefits observed in this controlled setting may overestimate those achievable in routine practice. The fixed-order design, with artificial intelligence-assisted consultations always conducted second, confounds findings with practice effects and scenario familiarity. Participants’ first exposure to the technology in a research context may also have generated novelty effects that would not necessarily persist with sustained use. The patient perspective in this study is that of trained standardized simulated patients, whose professionally informed observations should not be treated as equivalent to the lived experiences of individuals receiving psychiatric care. This constraint is particularly important for themes involving stigma, consent, privacy, and decision-making capacity. Finally, the research team’s prior knowledge of the positive quantitative findings from the parent simulation study may have influenced interpretation despite reflexive practices.

## 5 Conclusion

This exploratory qualitative study suggests that ambient AI scribes may support more humanistic, patient-centered psychiatric consultations by reducing documentation burden and sustaining clinician attention. Successful implementation requires attention to trust, transparent consent processes, clinician oversight, and the unique considerations of psychiatric practice, including stigma, privacy, and decision-making capacity. These findings provide qualitative depth to complement quantitative evidence and support further real-world evaluation of ambient AI scribes in psychiatric services.

## Data Availability

The original contributions presented in the study are included in the article/supplementary material. Further inquiries can be directed to the corresponding author.
